# Successive monitoring surveys of selected banned and restricted pesticide residues in vegetables from the northwest region of China from 2011 to 2013

**DOI:** 10.1186/s12889-017-4632-x

**Published:** 2017-08-02

**Authors:** Yan Yu, Senke Hu, Yuxuan Yang, Xiaodan Zhao, Jianjun Xue, Jinghua Zhang, Song Gao, Aimin Yang

**Affiliations:** 1 The first affiliated hospital of Xi’an Jiaotong University, No.277#, Yanta Road, Xi’an, Shaanxi 710061 China; 2 Xi’an Jiaotong University School of Medicine, No.76#, West Yanta Road, Xi’an, Shannxi 710061 China

**Keywords:** Vegetables, Pesticides, Residues, MRLs

## Abstract

**Background:**

A wide range of pesticides is applied for crop protection in vegetable cultivation in China. Regulation of pesticide maximum residue limits (MRLs) in vegetables is established but not fully enforced. And pesticide residues in vegetables were not well monitored. This study conducted the monitoring surveys from 2011 to 2013 to investigate the pesticides in vegetables in the northwest region of China.

**Methods:**

A multi-residue gas chromatography/mass spectrometry method (GC/MS) was used in determination of pesticides in vegetable samples. The χ^2^ test was used to compare the concentration of pesticide residues.

**Results:**

A total of 32 pesticide residues were detected in 518 samples from 20 types of vegetables in this study. 7.7% of the detected pesticide residues exceeded the MRLs. The percentages of residues that exceeded the MRLs for leafy, melon and fruit, and root vegetables were 11.2%, 5.1%, and 1.6%, respectively. There was no seasonal difference in the proportion of samples that exceeded the MRLs in different vegetables. A total of 84.3% (27/32) pesticides were detected at concentrations that exceeded MRLs. And of the 27 pesticides that exceeded the MRLs, 11 (40.7%) were banned for use in agriculture. The most frequently detected pesticides were Malathion (9.4%), Dichlorvos (8.7%), and Dimethoate (8.1%).

**Conclusion:**

The observed high rate of pesticides detected and high incidence of pesticide detection exceeding their MRLs in the commonly consumed vegetables indicated that the Good Agricultural Practices (GAP) may not be well followed. The management of pesticide use and control should be improved. Well-developed training programs should be initiated to improve pesticide application knowledge for farmers.

## Background

China is an agricultural country that plants a variety of vegetables. The majority of Chinese diets contain more than 300 g of vegetables in total meals per day [1]. In particular, a large proportion of vegetables are consumed in semi-processed or raw form. A wide range of pesticides is applied for crop protection in vegetable cultivation because of heavy pest infestation [2]. For example, organophosphorous, carbamate and pyrethroid are frequently used in China, especially on vegetables. Food commodity contamination from pesticide residues has become an important issue with growing concern in the world [3]. Studies have indicated that regulation of pesticide maximum residue limits (MRLs) in commodities is established but not fully enforced in many countries [4, 5]. In China, there are 433 types pesticides used in various foods with 4140 regulations of MRLs by the end of last year. In contrary, there are only 33 types of biological pesticides which are not banned or restricted [1]. Therefore, analysis of pesticide residues in food and other environmental commodities such as soil, water, fruit and total diet are essential for consumers. A few studies of monitoring pesticides have been conducted in China to assess the environmental load of these residues, and risk assessment for short-term intake of pesticide residues in crops has been carried out [2, 6–8]. However, ongoing monitoring of pesticide residues for vegetables in China is still scarce.

According to previous monitoring of pesticides used in vegetables [9, 10], 11 banned pesticides (Methylbenzene, Parathion, Parathion-methyl, Moncrotophos, Omethoate, Ethoprophos, Isazofos, Carbofuran, Methomyl, Kelthane and Fenvalerate) and 21 restricted pesticides (Phoxim, Phosmet, Dichlorvos, Dimethoate, Acephate, Chlorpyrifos, Fenitrothion, Trichlorfon, Malathion, Fenthion, Quinalphos, Methidathion, Ethion, Pirimicarb, Carbaryl, Metolcarb, Propoxur, Cypermethrin, Permethrin, Cyhalothrin and Deltamethrin) were included in this study.

This study is aimed to evaluate pesticide residues in the commonly consumed vegetables in the northwest region of China. And the findings will provide scientific evidence for Chinese agriculture authorities to improve the management of pesticide use and control, and to implement recommendations for reduction of pesticide residues and improvement of human and environmental safety.

## Methods

### Study design

A repeated cross-sectional study analyzing the presence of 32 pesticide residues in 20 types of vegetables on the 9 randomly selected markets in the northwest region of China was conducted in each year from 2011 to 2013.

Xi’an was chosen as a region to monitor the exposure of pesticide residues in vegetables because Xian city is the main center of vegetable trade in the northwest region of China. In 2011, the city was divided into four areas, namely, the east region, the west region, the south region and the north region. And two vegetable wholesale markets, three supermarkets and four trade markets were randomly sampled. In each market, 20 types of vegetables with large consumption in the northwest region was selected for monitoring, including leaf vegetables (baby cabbage, *Brassica chinensis*, cabbage, cauliflower, celery, Chinese cabbage, leek, lettuce, pakchoi, and spinach), melon and fruit vegetables (cowpea, cucumber, eggplant, green pepper, kidney bean, Pumpkin, and tomato), and root vegetables (carrot, potato, Ternip). In each month, new samples of 3–6 of 20 types vegetables in each market were collected from January 2011 to December 2013. Each sample was divided into two portions for detection, and the average value was used for data analysis.

### Sample treatment and detection

Referring to national standard methods [11, 12], the sample was first extracted using mixed acetone and dichloromethane in equal proportions as a solvent. Secondly, two solid-phase extraction methods were used to purify and concentrate the samples synchronously. Thirdly, GC and GC-MS were used to conduct qualitative and quantitative analyses.

**Fig. 1:**
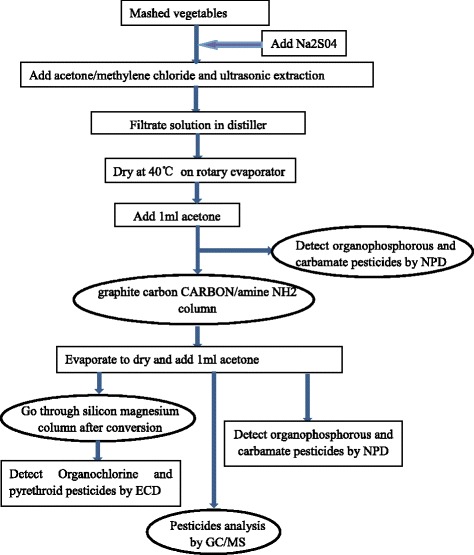
Process of samples treatment and detection

The sample treatment and detection procedure was applied as follows: (1) the edible portions of fresh vegetables were crushed and stirred, and 20 g was transferred to a 250 mL flask with a stopper; (2) 50 mL of acetone, 50 mL of methylene chloride and moderate anhydrous sodium sulfate were added to the flask, and ultrasonic extraction was conducted for 30 min; (3) the extraction solution was filtered using filter paper saturated with methylene chloride and subsequently purified using a graphite carbon carbon/amine NH2 column; (4) half of the cleaning fluid was dried on a rotary evaporator using a water bath at 45 °C, and the residue was washed several times with acetone. The acetone amount was fixed to 1 mL, and the sample was mixed and injected into the gas chromatograph to detect organophosphorous and carbamate pesticides via nitrogen phosphorous detector (NPD) or to detect 31 types of pesticides by GC/MS; (5) the other half of the cleaning fluid was used to conduct secondary purification using Florisl SPE via drying on a rotary evaporator using a water bath at 45 °C; (6) the residue was washed several times with acetone, the acetone capacity was fixed at 1 mL, and the sample was mixed and injected into the gas chromatograph to detect pyrethroid pesticides via electron capture detector (ECD). The details are shown in Fig. 1.

### Preparation of standard curve

An amount of 1 mL of each of 20 types of standard organophosphorus pesticide samples was added to a 10 mL volumetric flask. The pesticide standard solution volume was added at approximately 8 mL, the flask was placed on a nitrogen-blowing instrument, and the solution was slowly blown to approximately 3 mL. The next pesticide standard solution was added. Finally, acetone was used to formulate a 10 μg/mL mixed standard stock solution. Similarly, carbamate and pyrethroid pesticide standard solutions were also formulated to 10 μg/mL of a mixed standard stock solution. With progressive acetone dilution, the standard series were obtained at 0.100, 0.400, 1.00, 4.00 and 10.0 μg/mL.

### Quality control

Cabbage, leeks and carrots were taken as most representative leafy vegetables, bulb vegetables, and root vegetables to calculate the respective recovery rates. The standard pesticide reference samples were derived from the Ministry of Agriculture, Environmental Monitoring and Protection Center.

### Statistical analysis

All analyses were conducted using SPSS software (version 18.0, SPSS Inc., Chicago IL) with two-tailed *P* values, and *P* < 0.05 indicated statistical significance. Comparison of the concentration of pesticide residues was assessed using the χ^2^ test.

## Results

Using the gas chromatography/mass spectrometry method (GC/MS), this study analyzed the presence of 32 pesticide residues in vegetables on the market in the northwest region of China in consecutive years from 2011 to 2013. A total of 32 pesticides were detected with good sensitivity by the multi-residue analysis method, which was validated by evaluating the recovery, linearity and precision of peak areas (see Table 1). Good linearity was obtained with correlation coefficients of regression (R^2^ > 0.999) for each of the compound. The limit of detection (LOD) ranged from 0.002 to 0.01 mg/kg in this analysis.

**Table 1:** The Recovery, RSD, LOD, and LOQ of pesticides^a^

Pesticides	Recovery (%)	RSD (μg/mL)	R^2^	LOD (mg/kg)	LOQ (mg/kg)	MRL^b^ (mg/kg)
Acephate	87.5	0.04–20	0.9996	0.005	0.017	0.02
Carbaryl	89.3	0.10–20	0.9996	0.025	0.083	1
Carbofuran	89.8	0.10–20	0.9997	0.025	0.083	0.5–2
Chlorpyrifos	96.1	0.04–20	0.9995	0.005	0.033	0.5
Cyhalothrin	106.4	0.02–20	0.9994	0.005	0.017	0.2
Cypermethrin	93.2	0.02–20	0.9996	0.005	0.017	0.5
Decamethrin	101.7	0.02–20	0.9993	0.005	0.017	0.2
Dichlorvos	93.6	0.04–20	0.9995	0.005	0.033	0.02
Dicofol	106.9	0.04–20	0.9995	0.005	0.017	0.01–1
Dimethoate	104.7	0.04–20	0.9996	0.005	0.017	1
Ethion	93.8	0.04–20	0.9991	0.01	0.033	0.05
Ethoprophos	82.4	0.04–20	0.9994	0.01	0.017	0.01
Fenitrothion	88.5	0.04–20	0.9992	0.005	0.017	0.2
Fenthion	88.4	0.04–20	0.9994	0.01	0.017	0.05–1
Fenvalerate	109.1	0.02–20	0.9992	0.005	0.017	0.05
Isazofos	83.1	0.04–20	0.9991	0.005	0.017	0.03
Malathion	85.6	0.04–20	0.9994	0.01	0.017	0.5
Methidathion	86.6	0.04–20	0.9993	0.005	0.017	0.02
Methomyl	87.1	0.10–20	0.9991	0.025	0.083	1
Metolcarb	81.4	0.10–20	0.9995	0.025	0.083	0.05–1.0
Monocrotophos	93.7	0.04–20	0.9998	0.005	0.017	0.01
Omethoate	84.6	0.04–20	0.9997	0.005	0.033	0.02
Parathion	94.2	0.04–20	0.9996	0.005	0.033	0.05
Parathion-methyl	91.9	0.04–20	0.9997	0.01	0.033	0.1–0.5
Permethrin	92.7	0.02–20	0.9997	0.005	0.017	/
Phorate	90.2	0.04–20	0.9995	0.005	0.017	0.2–0.5
Phosmet	91.7	0.04–20	0.9992	0.005	0.017	0.5
Phoxim	89.4	0.04–20	0.9996	0.005	0.017	0.05
Pirimicarb	89.3	0.10–20	0.9995	0.025	0.083	0.02
Propoxur	76.9	0.10–20	0.9993	0.025	0.083	0.01
Quinalphos	91.6	0.04–20	0.9997	0.01	0.033	0.2
Trichlorfon	92.3	0.10–20	0.9995	0.01	0.017	0.01

^a^ *RSD* relative standard curve, *R* ^*2*^ correlation coefficient, *LOD* Limit of detection, *LOQ* Limit of quantification, *MRL* Maximum residue limit

^b^Refer to GB2763–2014 National food safety standard: Maximum residue limits for pesticides in food

7.7% of pesticide residues detected from vegetables exceeded the MRLs, and 26.7% of cowpea, 23.3% of celery, 16.7% of leeks, 14.3% of cabbage and 14.3% of *Brassica chinensis* contained pesticide residues that exceeded the MRLs, which were relatively higher compared with other vegetables (Table 2). Significant differences of the percentages of MRLs exceedance among leafy (11.2%), melon and fruit (5.1%), and root vegetables (1.6%) were observed (Table 3). The highest rate of samples with residues that exceeded the MRLs in three sampled sale sites occurred in trade markets with 13.1%, which was significantly higher than other two sites (Table 4).

**Table 2:** Summary of vegetables samples containing residues above MRLs

Vegetables	Samples	Number of samples				Samples with residues above MRLs	
		Total	2011	2012	2013	NO.	Rate (%)
Leafy vegetables	Baby cabbage	12	4	4	4	1	8.3
	*Brassica chinensis*	28	8	10	10	4	14.3
	Cabbage	28	8	10	10	4	14.3
	Cauliflower	25	6	9	10	2	8
	Celery	30	8	10	12	7	23.3
	Chinese cabbage	24	6	8	10	2	8.3
	Leek	30	8	10	12	5	16.7
	Lettuce	27	8	9	10	0	0
	Pakchoi	26	8	8	10	2	7.7
	Spinach	28	8	10	10	2	7.1
Melon and fruit vegetables	Cowpea	30	8	10	12	8	26.7
	Cucumber	30	8	10	12	2	6.7
	Eggplant	28	8	10	10	0	0
	Green pepper	28	8	10	10	0	0
	Kidney bean	28	8	10	10	0	3.6
	Pumpkin	26	8	8	10	0	0
	Tomato	26	8	8	10	0	0
Root vegetables	Carota	26	8	8	10	0	0
	Potato	26	8	8	10	0	0
	Ternip	12	4	4	4	1	8.3
	Total	518	148	174	196	40	7.7

**Table 3:** Pesticide residues in variety of vegetables

Vegetables	Samples	Samples with residues above MRL		χ^2^	*P*
		NO.	Rates (%)		
Leafy	258	29	11.2		
Melon And Fruit	196	10	5.1	9.907	0.007
Roots	64	1	1.6

**Table 4:** Pesticide residues in vegetables from different sale sites

Sale sites	Samples	Samples with residues above MRL		χ^2^	*P*
		NO.	Rates (%)		
Supermarket	246	9	3.7	12.031	0.002
Wholesale market	135	13	9.6		
Trade market	137	18	13.1		

As Table 5 shows, no statistically significant differences were observed in function of the date of production (season) neither in function of the seasonality of the vegetables. 9.3% of samples with residues above MRL were detected in conventional vegetables. In the 98 samples of organic vegetables, 1 pesticide (chlorpyrifos) was detected and was above the MRL in 1 sample (cucumber) collected from trade market in 2011.

**Table 5:** Pesticide residues in different seasons, and organic vs. conventional vegetables

Types	Sample numbers	Samples with residues above MRL		χ^2^	*P*
		NO.	Rates (%)		
Vegetables in winter and spring	224	13	5.8	2.038	0.153
Vegetables in summer and autumn	294	27	9.2		
Anti-seasonal Vegetables	124	12	9.7	0.875	0.350
Seasonal Vegetables	394	28	7.1		
Organic Vegetables	98	1	1.0	7.619	0.006
Conventional Vegetables	420	39	9.3		

A total of 27 pesticides were detected that exceeded the MRLs, and banned pesticides accounted for 40.7% (11/27) (Table 6). Out of 40 vegetable samples that exceeded the MRLs, banned pesticides were detected in 72.5% (29/40) of samples. The quantity of pesticide residues that exceeded the MRLs in 40 samples was 149, of which 49.0% (73/149) contained banned pesticides. The most frequently used pesticides were Malathion (9.4%), Dichlorvos (8.7%), and Dimethoate (8.1%).

**Table 6:** Distribution of 32 pesticide residues in all vegetables monitored

Pesticides	Maximum value detected (mg/kg)	Samples with residues above MRLs		Pesticides	Maximum value detected (mg/kg)	Samples with residues above MRL	
		NO.	%			NO.	%
Malathion	2.256	14	9.4	Dicofol	7.129	4	2.6
Dichlorvos	2.226	13	8.7	Trichlorfon	1.546	3	2
Dimethoate	4.496	12	8.1	Fenvalerate	0.856	3	2
Omethoate	3.922	10	6.7	Acephate	2.542	2	1.3
Carbofuran	0.186	9	6.1	Methidathion	0.038	2	1.3
Ethoprophos	0.028	9	6	Quinalphos	0.468	2	1.3
Isazofos	0.031	8	5.4	Propoxur	5.566	2	1.3
Monocrotophos	0.11	8	5.4	Pirimicarb	1.858	2	1.3
Metolcarb		8	5.4	Permethrin	2.566	2	1.3
Cypermethrin	1.738	7	4.7	Cyhalothrin	1.438	2	1.3
Phorate	1.432	6	4	Fenitrothion	0.13	1	0.7
Parathion-methyl	0.884	6	4	Fenthion	0.03	0	0
Parathion	1.658	4	2.7	Phoxim	0.025	0	0
Chlorpyrifos	1.884	4	2.7	Phosmet	0.168	0	0
Ethion	0.082	4	2.7	Carbaryl	0.28	0	0
Methomyl	3.974	2	2.7	Deltamethrin	0.045	0	0

The pesticides were detected in selected vegetables and up to five types of pesticides were detected exceeding the MRLs in the selected vegetables (Table 7).

**Table 7:** Pesticides detected in selected vegetables

Vegetables	No. of samples	No. of samples exceeding the MRLs	Name of pesticides detected above the MRLs
Spinach	28	2	(1):Dichlorvos, Ethoprophos
			(2):Malathion
Leek	30	5	(1):Dichlorvos, Malathion, Omethoate, Trichlorfon
			(2):Dichlorvos, Dimethoate, Isazofos, Fenvalerate
			(3):Malathion, Dimethoate, Monocrotophos
			(4):Dimethoate, Ethoprophos, Cypermethrin
			(5):Omethoate, Carbofuran, Phorate
Cabbage	28	4	(1):Dichlorvos, Ethoprophos, Trichlorfon, Acephate
			(2):Dichlorvos, Omethoate, Monocrotophos
			(3):Malathion, Metolcarb, Dicofol
			(4):Dimethoate, Isazofos, Chlorpyrifos
Celery	30	7	(1):Dichlorvos, Malathion, Ethoprophos, Cypermethrin, Parathion
			(2):Dichlorvos, Dimethoate, Carbofuran, Parathion-methyl
			(3):Cypermethrin, Parathion-methyl, Phorate, Parathion, Methidathion
			(4):Dichlorvos, Omethoate, Isazofos, Ethion
			(5):Malathion, Dimethoate, Monocrotophos, Parathion, Cyhalothrin
			(6):Dimethoate, Carbofuran, Cypermethrin, Ethion
			(7):Omethoate, Metolcarb, Parathion-methyl, Dicofol, Methidathion
Green pepper	28	4	(1):Dichlorvos, Metolcarb
			(2):Malathion, Fenvalerate
			(3):Dimethoate, Monocrotophos
			(4):Metolcarb, Propoxur
Cauliflower	25	2	(1):Dichlorvos, Isazofos, Propoxur, Permethrin
			(2):Malathion, Ethoprophos, Carbofuran
Chinese cabbage	24	2	(1):Malathion, Metolcarb, Monocrotophos, Fenitrothion
			(2):Chlorpyrifos, Isazofos, Dicofol, Methomyl
Pakchoi	26	2	(1):Omethoate, Carbofuran, Ethion
			(2):Malathion, Metolcarb, Cypermethrin
Baby cabbage	12	1	(1):Malathion, Ethoprophos, Monocrotophos, Parathion-methyl, Methomyl
Cucumber	30	2	(1):Dichlorvos, Carbofuran, Metolcarb, Isazofos
			(2):Malathion, Ethoprophos, Phorate, Pirimicarb
Cowpea	30	8	(1):Dichlorvos, Malathion, Omethoate, Cypermethrin, Ethion, Fenvalerate
			(2):Dichlorvos, Dimethoate, Carbofuran, Phorate, Chlorpyrifos, Acephate
			(3):Omethoate, Ethoprophos, Parathion-methyl, Trichlorfon
			(4):Malathion, Dimethoate, Monocrotophos, Phorate, Pirimicarb
			(5):Dimethoate, Carbofuran, Cypermethrin, Parathion, Quinalphos
			(6):Dimethoate, Ethoprophos, Parathion-methyl, Chlorpyrifos
			(7):Omethoate, Carbofuran, Monocrotophos, Dicofol, Cyhalothrin
			(8):Phorate, Omethoate, Isazofos, Quinalphos, Permethrin
Ternip	12	1	(1):Metolcarb, Isazofos
Total	518	40	149

## Discussion

In our study, 32 pesticides were detected with good sensitivity using a multi-residue analysis method validated by evaluating recovery, linearity, and precision. Good linearity was observed for all of the compounds with excellent correlation coefficients of regression (R^2^ > 0.999). Good recoveries were obtained for all pesticides detected, and all indicators in Table 1 showed that the results were reliable for analysis of pesticide residues.

To our knowledge, this study is first to determine the levels of 32 pesticide residues on various vegetables frequently consumed in northwest region of China. In China, 72% of pesticides used in crops, especially in vegetables, consist of organic phosphorus materials, including parathion, parathion-methyl, monocrotophos, ethoprophos, omethoate, phorate, malathion, isazofos, carbofuran, dicofol and cyhalothrin [13], all of which were detected in this study. This indicates that pesticides are used widely and extensively in the vegetables cultivation in northwest region of China. And 72.5% of samples exceeding the MRLs contained banned pesticides, which suggested that banned pesticides might still be frequently used in vegetables. All of these results indicate a high incidence of pesticide residues in collected vegetable samples, similar to contamination reported in other region of China and other countries, especially in developing countries [14–17]. These results reflect that a certain proportion of farmers may not follow Good Agricultural Practices (GAP) in China. Future studies may be conducted to trace the farms where the vegetables are grown and to explore the factors especially farmers’ behaviors that contribute to the MRL exceedance. And the government should initiate a well-developed program on farmers’ participatory training to enhance farmers’ knowledge of pests and non-chemical alternatives and management options as other studies showed [18, 19].

The pesticides were detected in all samples collected from all the 3 local markets investigated, and the incidence of detected pesticides over MRLs varied among the markets. Vegetables in supermarkets generally originate from a large vegetable production base with strict use of pesticides. Additionally, supermarkets have full-time staff responsible for detecting pesticide residues in vegetables to ensure food safety. In contrast, vegetables in wholesale markets and trade market mostly come from scattered vegetable farmers who lack awareness of scientific and rational pesticide use [20]. 1 sample of organic foods was detected with pesticide from trade market. Although standards of organic foods may vary worldwide, synthetic pesticides are strictly banned in organic foods. But in this study, chlorpyrifos was detected in cucumber from organic farming in 2011. The possible reason may be related to the sources (or farms) contamination [21]. Although no organic vegetables samples with pesticide residues were detected in the following 2 years, monitoring and supervision on the organic vegetables should be strengthened.

No significant difference of pesticide residues was observed between anti-seasonal vegetables and seasonal vegetables, which showed a similar quantity of pesticides use in each season. Bhanti et al. found the winter vegetables are the most contaminated followed by summer and rainy vegetables by analyzing the pesticide residue concentrations in vegetables of different seasons [22]. For the specific type of vegetables, a relatively high rate of samples with residues that exceeded the MRLs appeared in cowpea, celery, leeks, cabbage and Brassica, which might suggest that these vegetables were more susceptible to pests and had large contact areas with pesticides [20]. It is urgent to strengthen monitoring and supervision in these vegetables in the northwest region of China.

More than one residue was detected in certain vegetable samples, especially the vegetables highly sensitive to pests that require multiple applications of pesticides, such as celery, cauliflower, and Chinese cabbage. During recent years, because of pest resistance, multiple pesticides have been widely applied for planting of vegetables [19, 23]. Multiple pesticides contamination has been reported by several studies [24, 25]. In this study, residues of 2–5 pesticides were observed in each of 39 samples of 12 vegetable types, indicating mixed use of pesticides in planting vegetables. However, food hygiene standards about multiple pesticides were still not established in China.

## Conclusions

This study provides scientific evidences of detected residues of many pesticides in the commonly used vegetables in the northwest region of China. The important findings are the observed high rate of pesticides detected and high incidence of pesticide detection exceeding their MRLs in the commonly consumed vegetables, indicating the GAP may not be well followed. Attention should be paid for Chinese agriculture authorities to improve the management of pesticide use and control. In addition, well-developed training programs should be initiated to improve pesticide application knowledge for farmers. And monitoring of pesticide residues in vegetables should be performed on a routine basis. The results are also helpful for the risk assessment of consumers’ exposure to those pesticide residues. Consumers should wash vegetables with more running water before use, which will reduce the intake of pesticides.

## Abbreviations

ECD: Electron capture detector
GAP: Good Agricultural Practices
GC/MS: Gas chromatography/mass spectrometry method
LOD: The limit of detection
MRLs: Maximum residue limits
NPD: Nitrogen phosphorous detector
